# Cortical Auditory Event-Related Potentials and Categorical Perception of Voice Onset Time in Children With an Auditory Neuropathy Spectrum Disorder

**DOI:** 10.3389/fnhum.2020.00184

**Published:** 2020-05-25

**Authors:** Tyler C. McFayden, Paola Baskin, Joseph D. W. Stephens, Shuman He

**Affiliations:** ^1^Department of Psychology, Virginia Polytechnic Institute and State University, Blacksburg, VA, United States; ^2^Department of Anesthesiology, School of Medicine, University of California, San Diego, San Diego, CA, United States; ^3^Department of Psychology, North Carolina Agricultural and Technical State University, Greensboro, NC, United States; ^4^Department of Otolaryngology—Head and Neck Surgery, Wexner Medical Center, The Ohio State University, Columbus, OH, United States; ^5^Department of Audiology, Nationwide Children’s Hospital, Columbus, OH, United States

**Keywords:** auditory neuropathy spectrum disorders, voice onset time, auditory event-related response, speech perception, categorical perception

## Abstract

**Objective**: This study evaluated cortical encoding of voice onset time (VOT) in quiet and noise, and their potential associations with the behavioral categorical perception of VOT in children with auditory neuropathy spectrum disorder (ANSD).

**Design**: Subjects were 11 children with ANSD ranging in age between 6.4 and 16.2 years. The stimulus was an /aba/-/apa/ vowel-consonant-vowel continuum comprising eight tokens with VOTs ranging from 0 ms (voiced endpoint) to 88 ms (voiceless endpoint). For speech in noise, speech tokens were mixed with the speech-shaped noise from the Hearing In Noise Test at a signal-to-noise ratio (SNR) of +5 dB. Speech-evoked auditory event-related potentials (ERPs) and behavioral categorization perception of VOT were measured in quiet in all subjects, and at an SNR of +5 dB in seven subjects. The stimuli were presented at 35 dB SL (re: pure tone average) or 115 dB SPL if this limit was less than 35 dB SL. In addition to the onset response, the auditory change complex (ACC) elicited by VOT was recorded in eight subjects.

**Results**: Speech evoked ERPs recorded in all subjects consisted of a vertex positive peak (i.e., P1), followed by a trough occurring approximately 100 ms later (i.e., N2). For results measured in quiet, there was no significant difference in categorical boundaries estimated using ERP measures and behavioral procedures. Categorical boundaries estimated in quiet using both ERP and behavioral measures closely correlated with the most-recently measured Phonetically Balanced Kindergarten (PBK) scores. Adding a competing background noise did not affect categorical boundaries estimated using either behavioral or ERP procedures in three subjects. For the other four subjects, categorical boundaries estimated in noise using behavioral measures were prolonged. However, adding background noise only increased categorical boundaries measured using ERPs in three out of these four subjects.

**Conclusions**: VCV continuum can be used to evaluate behavioral identification and the neural encoding of VOT in children with ANSD. In quiet, categorical boundaries of VOT estimated using behavioral measures and ERP recordings are closely associated with speech recognition performance in children with ANSD. Underlying mechanisms for excessive speech perception deficits in noise may vary for individual patients with ANSD.

## Introduction

Auditory neuropathy spectrum disorder (ANSD) is a form of hearing impairment characterized by normal outer hair cell function, as indicated by the presence of cochlear microphonics (CMs) and/or otoacoustic emissions (OAE), in conjunction with an aberrant auditory neural system, as revealed by an absent or abnormal auditory brainstem response (ABR). ANSD is estimated to be present in 5–10% of newly identified hearing loss cases each year (Rance, 2005; Vlastarakos et al., 2008; Berlin et al., 2010; Bielecki et al., 2012). The presence of ANSD has been linked to several risk factors including premature birth, neonatal distress (e.g., hyperbilirubinemia, anoxia, artificial ventilation), infection (e.g., mumps, meningitis), neuropathic disorders (e.g., Charcot-Marie-Tooth syndrome, Friedreich’s Ataxia), genetic factors (e.g., mutations in the otoferlin gene), and ototoxic drugs (e.g., carboplatin; Rance et al., 1999; Madden et al., 2002). Cases of ANSD with no apparent risk factors or medical comorbidities have also been reported (Berlin et al., 2010; Teagle et al., 2010; Roush et al., 2011; Bielecki et al., 2012; Pelosi et al., 2012). The exact lesions underlying the pathophysiology of ANSD have yet to be determined. Proposed sites of lesions include, but are not limited to, cochlear inner hair cells, synapses between inner hair cells and Type I auditory nerve fibers, and synapses between neurons in the auditory pathway.

Patients with ANSD typically exhibit poorer speech perception capability than would otherwise be expected based on the degree of hearing loss (Rance, 2005). These speech perception deficits can be partially accounted for by an impaired ability of the auditory system to detect changes in stimuli over time (i.e., temporal processing). Results of previous studies have shown that patients with ANSD have temporal processing deficits, and the severity of the deficits strongly correlates with their speech perception abilities (Starr et al., 1991; Zeng et al., 1999, 2001, 2005; Michalewski et al., 2005; Rance, 2005; He et al., 2015). Also, patients with ANSD typically experience excessive difficulty in understanding speech in the presence of competing for background noise (Kraus et al., 1984, 2000; Shallop, 2002; Zeng and Liu, 2006; Rance et al., 2007; Berlin et al., 2010). For example, Kraus et al. (2000) reported a case of an adult patient with ANSD who exhibited 100% speech recognition of monosyllabic words in quiet but achieved only 10% correct recognition of words at a signal-to-noise ratio (SNR) of +3 dB. In contrast, normal hearing subjects were able to retain an average score of 40% at the same SNR. This case was unique in that the patient had normal hearing thresholds, which indicated that her perceptual difficulties did not stem from diminished audibility, but rather from ANSD-associated neural pathophysiology such as neural dyssynchrony. Similarly, Shallop (2002) presented an adult female subject who scored 100% on a speech perception sentence test in quiet but was unable to correctly identify any sentences in noise conditions of +12 dB SNR, despite hearing thresholds indicating only mild to moderate hearing loss. To date, the underlying mechanisms of this excessive difficulty in understanding speech with background noise remain poorly understood for patients with ANSD.

Cortical auditory event-related potentials (ERPs), including the onset response and the auditory change complex (ACC), are neural responses generated at the auditory cortex that can be recorded from surface electrodes placed on the scalp. The onset of ERP is elicited by the onset of sound, and its presence indicates sound detection. The ACC is elicited by a stimulus change that occurs within an ongoing, long-duration signal, and its presence provides evidence of auditory discrimination capacity at the level of the auditory cortex (Martin et al., 2008). Previous animal studies have demonstrated that auditory cortex is an important contributor to signal-in-noise encoding (Phillips, 1985, 1990; Phillips and Hall, 1986; Phillips and Kelly, 1992). Robust ERP responses are recorded only if the listener possesses accurate neural synchronization in response to sound. Maintaining synchronized neural responses that are time-locked to the speech stimuli is critical for successful speech perception in quiet and noise (Kraus and Nicol, 2003). Therefore, using ERPs to examine the neurophysiological representation of speech sounds in quiet and competing background noise at the level of the auditory cortex will be extremely beneficial for better understanding and characterizing speech perception difficulties in patients with ANSD.

In natural speech, the voice onset time (VOT) is a temporal cue that is crucial for differentiating voiced and voiceless English stop consonants. VOT refers to the interval between the release of a stop consonant (the burst) and the beginning of vocal fold vibration or voicing onset (Lisker and Abramson, 1964). Voiced stop consonants (e.g., /ba/, /da/, /ga/) have a relatively short VOT (generally 0–20 ms) and voiceless stop consonants (e.g., /pa/, /ta/, and /ka/) have a longer VOT. As the VOT increases, the perception rapidly changes from a voiced stop consonant to a voiceless consonant at 20–40 ms. The abrupt change in consonant identification is a classic example of categorical speech perception. Acuity for VOT identification is highly dependent on the synchronized neural response evoked by the onset of voicing (Sinex and McDonald, 1989; Sinex et al., 1991; Sinex and Narayan, 1994).

ERP measures have previously been used to objectively evaluate neural encoding of VOT at the level of the auditory cortex in normal-hearing (NH) listeners and cochlear implant users (Sharma and Dorman, 1999, 2000; Sharma et al., 2000; Steinschneider et al., 2003, 2005, 2013; Roman et al., 2004; Frye et al., 2007; Horev et al., 2007; King et al., 2008; Elangovan and Stuart, 2011; Dimitrijevic et al., 2012; Apeksha and Kumar, 2019). For example, King et al. (2008) used ERPs to investigate the underlying neural mechanisms of categorical speech perception in NH children. The results of this study showed prolonged P1 latency recorded at long VOTs. Morphological characteristics of ERPs measured at different VOTs were the same. However, using the same stimuli, Sharma and Dorman (1999) observed an extra peak in ERP responses at long VOTs in normal hearing (NH) adults and this extra peak is believed to be elicited by the onset of voicing (Steinschneider et al., 2003, 2005, 2013). It should be pointed out that speech tokens used in these two studies were synthetic stop consonant-vowel (CV) syllables with a relatively short duration: 200 ms. Studies have shown that neural generators of ERPs have longer recovery periods in children than in adults (Ceponiene et al., 1998; Gilley et al., 2005). Therefore, the lack of the extra peak evoked by VOT in children could be due to insufficient separation between the stimulus onset and VOT such that responses evoked by the syllable onset and VOT overlap, resulting in a single broad peak in children as observed in King et al. (2008). To address this potential issue, relatively long vowel-consonant-vowel (VCV) stimuli that contain VOTs were used in this study so that neurons would have sufficient recovery time after responding to the syllable onset.

In summary, patients with ANSD are known to have neural dyssynchrony. Theoretically, the neurophysiological encoding of VOT in these patients should be compromised, which should account for their impaired categorical perception of VOT (Rance et al., 2008). However, this predictive framework has not been systematically evaluated in the pediatric ANSD population. Furthermore, the neural encoding of speech stimuli in noise at the level of the auditory cortex and the association between neural encoding and behavioral categorical perception of VOT in quiet and noise in subjects with ANSD remains largely unknown. To address these needs, this study investigated the behavioral categorical perception of VOT and ERPs evoked by VOT in children with ANSD in quiet and speech-shaped background noise. We hypothesized that: (1) the precision of neural encoding of VOT would affect behavioral categorical perception performance in children with ANSD, and (2) ERPs evoked by the VOT in children with ANSD would be adversely affected by competing for noise.

## Materials and Methods

### Subjects

Study participants included 11 children with ANSD (S1–S11) ranging in age between 6.4 and 16.2 years (mean: 10.1 years, SD: 3.0 years, *n* = 5 females). All of the subjects were clinically diagnosed with ANSD based on the gross inconsistency between cochlear and neural functions. Specifically, subjects with ANSD were diagnosed based on the presence of CM (±OAE) with absent or abnormal ABRs. Results of Magnetic Resonance Imaging (MRI) revealed no evidence of dysplasia of the inner ear or internal auditory canal in any of these subjects. None of the subjects had any known cognitive impairments or developmental delays that might affect the results of this study. All subjects were placed in mainstream classrooms. For all except for one subject (S8), English was the only language used in their families. S8 was learning English as her primary language in school and used a combination of English and Hebrew at home.

The ear with better pure-tone hearing thresholds was selected as the test ear in this study. The degree of hearing loss of the test ear ranged from normal to severe, with an average pure tone audiometric threshold of 54.2 dB HL (calculated as the average of thresholds at 0.5, 1.0, and 2.0 kHz for each patient). All subjects except for S6 were fitted with hearing aids in the ears tested in this study. S6 did not use any amplification at the time of testing. Detailed demographic and audiology information for these subjects are listed in Table 1.

**Table 1:** Demographic information of all subjects with auditory neuropathy spectrum disorder (ANSD) who participated in this study.

Subject number	Gender	Risk factor	Ear tested	Age at testing (years)	3-Frequency PTA (dB HL)	PBK word scores (%)
1	F	Prematurity, hyperbilirubinemia	R	7.6	53.33	64.00
2	M	None	R	9.4	61.7	76.00
3	M	None	R	12.5	85	48.00
4	M	Prematurity	R	13.3	43.3	96.00
5	F	None	R	16.2	70	68.00
6	M	Prematurity	L	8.5	26.7	76.00
7	F	Prematurity, hyperbilirubinemia	L	10.5	71.7	40.00
8	F	Prematurity	R	6.4	66.7	28.00
9	M	Prematurity, hypoxia	L	7.8	56.7	16.00
10	M	Prematurity	L	6.6	55	36.00
11	F	None	R	11.12	60	80.00

*PTA, Pure-Tone Average; PBK, Phonetically Balanced Kindergarten*.

All subjects were recruited from the Ear & Hearing Center within the Department of Otolaryngology/Head and Neck Surgery at the University of North Carolina at Chapel Hill (UNC-CH). This study was approved by the Institutional Review Board (IRB) at UNC-CH. Written consent for the study procedures was provided by legal guardians of all subjects. Written assent for the study procedures was obtained from all subjects except for subjects 8 and 10. Oral assent for participating in this study was provided by these two subjects. Monetary compensation was provided to all subjects for participating in the study.

### Stimuli

The stimuli were a VCV continuum with the consonants ranging from a voiced /b/ to a voiceless /p/, in an /a/ context. For this study, the goal of the stimulus creation process was to produce a natural-sounding series in which VOT was manipulated while holding other acoustic/phonetic properties constant to the extent that such control was possible. In other studies that use voicing continua based on natural speech, stimuli are often created *via* cross-splicing between recordings of endpoint tokens (e.g., Ganong, 1980). However, VOT is only one of several acoustic/phonetic properties that may differ between natural tokens of voiced and voiceless stop consonants in English (Lisker, 1986). Perceptual judgments of voiced and voiceless consonants can be influenced by many differences, for example, variations in the first formant (F1) onset shift perception of voicing in addition to VOT (Stevens and Klatt, 1974). Thus, the current methods attempted to isolate the VOT manipulation by using a re-synthesis procedure instead of cross-splicing.

Stimuli were created based on materials and methods used in a previous study (Stephens and Holt, 2011) that manipulated VCV utterances by using linear predictive coding (LPC; Atal and Hanauer, 1971) to derive source and filter properties of natural utterances and resynthesize intermediate utterances based on modifications to the source and/or filter properties. For the current stimuli, Praat software (Boersma, 2001) was used to derive LPC filter coefficients from a natural token of /aba/. A separate natural token of /ada/, temporally aligned with the /aba/ utterance, was inverse filtered by its LPC coefficients to produce a voicing source, which was then edited to create eight different voicing sources with varying VOT. The series of source waveforms were created by deleting pitch periods from the original source and inserting equivalent lengths of Gaussian noise at the onset of the consonant to simulate aspiration. All other aspects of the voicing sources were held constant.

**Figure 1:**
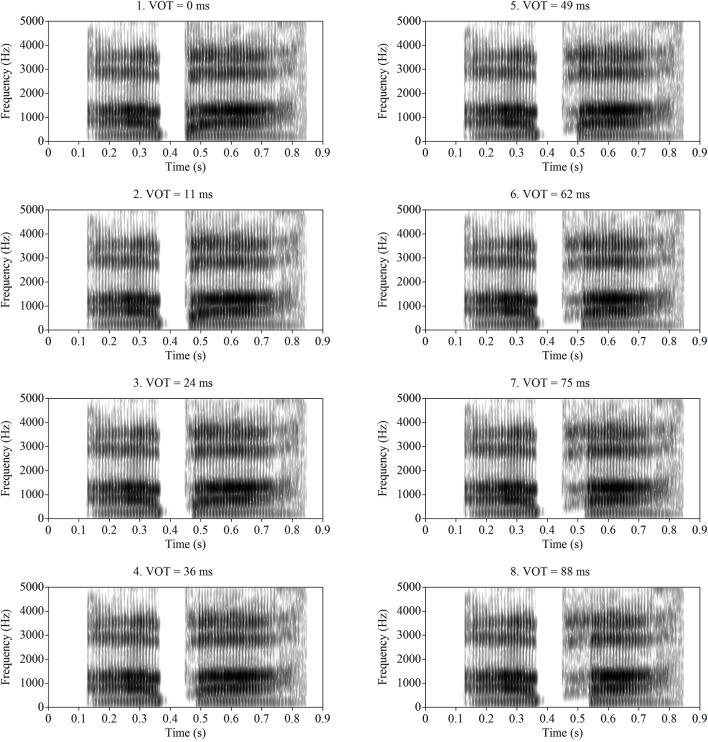
Spectrogram of the /aba-apa/ continuum used in this study. Voice onset times (VOTs) are used to label these figures.

The LPC filter for /aba/ was then applied to each of the eight source signals to create sounds with identical formant structure to /aba/, but with varying VOTs. The resulting VOTs in the resynthesized stimuli were 0 ms (voiced endpoint), 11 ms, 24 ms, 29 ms, 36 ms, 49 ms, 62 ms, 75 ms, and 88 ms (voiceless endpoint). The slight variation in step size along the series resulted from the procedure of lengthening VOT by deleting pitch periods from the voicing source. On average, pitch periods in the signal were roughly 10 ms, corresponding to a fundamental frequency of approximately 100 Hz; however, there was some natural variation in the length of individual pitch periods. All sounds were sampled at 11,025 Hz and were matched in RMS amplitude after the re-synthesis procedure. The overall length of each stimulus was approximately 720 ms. Spectrograms of each of the eight stimuli are displayed in Figure 1. As shown in the figure, the onset of periodicity gradually shifted from one end of the series to the other, while the formant structure remained relatively constant. In the longer-VOT stimuli, the formant frequencies (including F1) were excited by the aspiration noise that was inserted into the voicing sources (i.e., F1 was not cut back). It should be noted that the F1 and F2 frequencies *at the onset* of voicing were slightly different at different VOTs. In perception, these spectral differences immediately following voice onset may also contribute to the perception of consonant voicing, but systematic experiments with synthetic stimuli have found formant frequency at voice onset to be a relatively weaker cue than VOT (Lisker, 1975). Furthermore, formant frequency differences have been found to influence consonant voicing distinctions to a lesser extent in children (the population of interest in the current study), than in adults (Morrongiello et al., 1984). More importantly, altering formant frequency has been found to shift ERP response patterns and behavioral categorical perception of VOT in a parallel manner (Steinschneider et al., 2005). Nevertheless, stimuli used in this study did not solely contain timing cues, which needs to be taken into consideration when interpreting the results of this study.

For VOT identification in noise, speech tokens were mixed with the speech-shaped noise taken from the Hearing In Noise Test for Children (HINT-C; Nilsson et al., 1996) at an SNR of +5 dB using Audacity (v.1.3 Beta).

### General Procedures

Each subject completed both behavioral measures of categorical perception of VOT and electrophysiological recordings of ERPs for all stimulation conditions in which they were tested. Both procedures were undertaken *without* using subjects’ hearing aids. These two measures were completed in different sessions scheduled on the same day. In general, it took about three and a half hours to complete both procedures for each test condition (i.e., quiet or noise).

Categorical perception of VOT and electrophysiological measures of the speech-evoked ERP were evaluated in quiet in all subjects. Besides, these two procedures were also undertaken at an SNR of +5 dB in seven subjects (S1–S5, S8, and S11). ERPs evoked by stimuli presented in noise with VOTs of 75 or 88 ms were not recorded in S11 due to time constraints. All stimuli were presented using the Neuroscan Stim2 (Compumedics, Charlotte, NC) at 35 dB SL (re: pure tone average) or 115 dB SPL (maximum output level of the stimulation system without any distortion) if this limit was less than 35 dB SL. Stimulus level was calibrated using a Larson-Davis 824 sound level meter, a 6-cc coupler for supra-aural earphone, and a 2-cc coupler for the ER-3A insert earphone.

#### Categorical Perception of VOT

The stimulus was delivered through a Sennheiser supra-aural earphone (HD8 DJ). A two-interval, two-alternative forced-choice procedure was used. Listening intervals were visually indicated using computer graphics with /aba/ and /apa/ shown in yellow and blue, respectively. An initial practice session using tokens with VOTs of 0 and 88 ms was provided to each subject before data collection. Ten presentations of each of eight tokens (i.e., 80 in total) were presented to the test ear. The sequence of these presentations was pseudo-randomized on a trial-by-trial basis. For each presentation, subjects were asked to indicate whether they heard /aba/ or /apa/ by pointing or selecting the interval with the associated graphic or color. No feedback was provided. The percentage of /aba/ responses was calculated. Subjects needed approximately 10 min to complete this task for each stimulation condition.

#### Electrophysiological Recordings

For each subject, electrophysiological recordings were completed in up to four test sessions and each session lasted approximately 2 h. Subjects were tested in a single-walled sound booth. They were seated in a comfortable chair watching a silent movie with closed captioning. Breaks were provided as necessary. All stimuli were presented through an ER-3A insert earphone. The inter-stimulus interval was 1,200 ms.

Electroencephalographic (EEG) activity was recorded using the Neuroscan SCAN 4.4 software and a SynAmpRT amplifier (Compumedics, Charlotte, NC, USA) with a sampling rate of 1,000 Hz. Disposable, sterile Ag-AgCl surface recording electrodes were used to record the EEG. In nine subjects, responses were recorded differentially from five electrodes (Fz, FCz, Cz, C3, and C4) to contralateral mastoid (A_1/2_, reference) relative to body ground at the low forehead (F_pz_). However, S9 pulled off three electrodes placed on the scalp (i.e., FCz, Cz, and C4) before data collection was completed. Replacing these scalp electrodes was rejected by this subject. Responses were only differentially recorded from Fz to contralateral mastoid in S8 and S10 due to the lack of sufficient subject compliance for the prolonged testing time required by this study. Therefore, ERP responses were only recorded from Fz for all stimulation conditions in these three subjects. Eyeblink activity was monitored using surface electrodes placed superiorly and inferiorly to one eye. Responses exceeding ±100 μV were rejected from averaging. Electrode impedances were maintained below 5 kΩ for all subjects. The order in which the EEG data were collected was randomized across VOTs to minimize the potential effect of attention or fatigue on EEG results. The EEG was epoched and baseline corrected online using a window of 2,000 ms, including a 100-ms pre-stimulus baseline and a 1,900-ms peri/post-stimulus time. Auditory evoked responses were amplified and analog band-pass filtered online between 0.1 and 100 Hz (12 dB/octave roll-off). After artifact rejection, the remaining (at least 100) artifact-free sweeps were averaged to yield one replication. For each stimulation condition for each subject, three replications with 100 artifact-free sweeps/replication were recorded at all recording electrode locations. These recordings were digitally filtered between 1 and 30 Hz (12 dB/octave roll-off) offline before response identification and amplitude measurements.

### Data Analysis

For the results of the behavioral categorical perception test, the percentage of /apa/ responses were calculated and plotted as a function of VOT. These results were fitted using a least-squares procedure for each subject with a logistic function of the form:

$$P(x)=a/(1+e^{-b(x-x_{0})})$$

where *P* is the percentage of trials the token was perceived as /apa/ (0–1), *a* is the upper limit of the performance, *x_0_* is the midpoint of the function, and *b* is the slope, where larger values represent steep functions.

The point on the psychometric function that corresponds to chance performance (the token was perceived to be /apa/ 50% of the time) was determined for each subject. The VOT that yielded this chance performance was defined as the behavioral categorical boundary of the /aba-apa/ continuum. This criterion has been used in previously published studies (e.g., Sharma and Dorman, 1999; Elangovan and Stuart, 2008).

Grand mean averages of ERPs recorded from all subjects were computed for each stimulating condition and used to determine the latency ranges for which the onset and the ACC response were measured. The windows for the onset and the ACC response were from 25 to 240 ms and from 390 to 640 ms, relative to the stimulus onset, respectively. For each subject, replications evoked by the same speech tokens were averaged for each stimulation condition. As a result, eight averaged responses were yielded for each subject in each stimulation condition except for S11 tested at the SNR of 5 dB. The averaged responses were used for peak identification, as well as peak amplitude and latency measures. For the eight subjects from whom ERP responses were recorded at five recording electrode locations (i.e., S1–S7, S11), responses were examined across these electrode sites to help identify ACC responses. ERP responses recorded in these subjects were independently assessed by two researchers (authors PB and SH). The presence of the ACC response was determined based on two criteria: (1) a repeatable neural response within the expected time window for the ACC based on mutual agreement between the two researchers; and (2) an ACC response recorded in all five electrode sites. For three subjects whose ERPs were only recorded from Fz, their responses were evaluated by the third researcher (author TCM). The presence of the ACC response in these three cases was determined based on mutual agreement among all three researchers that a repeatable neural response could be identified within the expected time window for the ACC. The objective categorical boundary was defined as the shortest VOT that could reliably evoke the ACC response in this study. For the ACC response identification, the inter-judge agreement among three researchers was 91% and between authors, PL and SH were 93%. In cases where judges initially differed in peak identification, the differences were mutually resolved following consultation and discussions.

Despite a wide range of ages at the time of testing, both the onset response and the ACC recorded in all subjects consisted of a vertex positive peak (P1) followed by a negative trough (N2). For the onset and the ACC, the P1 was identified as a positive peak occurring within a time window between 40 and 150 ms and a time window between 410 and 520 ms after stimulus onset, respectively. The N2 was identified as the negative trough following the P1 occurring approximately 100 ms later. Latencies and amplitudes of the P1 and N2 peaks were measured using a custom-designed MATLAB (Mathworks) software at the maximum positivity or negativity in the estimated latency window of both peaks. The peak-to-peak amplitude was measured as the difference in voltage between the P1 and N2 peaks.

Dependent variables measured for ERP results included the objective categorical boundary, latencies of the P1 and the N2 peak, and the peak-to-peak amplitude of the onset and the ACC responses. The Friedman test was used to evaluate the effects of recording electrode location on ERP responses in a subgroup of seven subjects. The related-sample Wilcoxon Signed Rank test was used to compare: (1) behavioral and objective categorical boundaries; and (2) effects of competing for noise on amplitude and latency of P1 and N2 peaks of the onset and the ACC responses for a subgroup of seven subjects. The one-tailed Spearman Rank correlation test was used to evaluate the association between behavioral and objective categorical boundaries. Also, potential associations between categorical boundaries and the most recently measured aided Phonetically Balanced Kindergarten (PBK) word scores were evaluated using a one-tailed Spearman Rank Correlation test for these subjects. The PBK word scores were measured approximately 1 month before the study for all subjects.

## Results

Overall, behavioral categorical perception of VOT and speech evoked ERPs were measured from subjects in both quiet and noise conditions.

### Results Measured in Quiet

**Figure 2:**
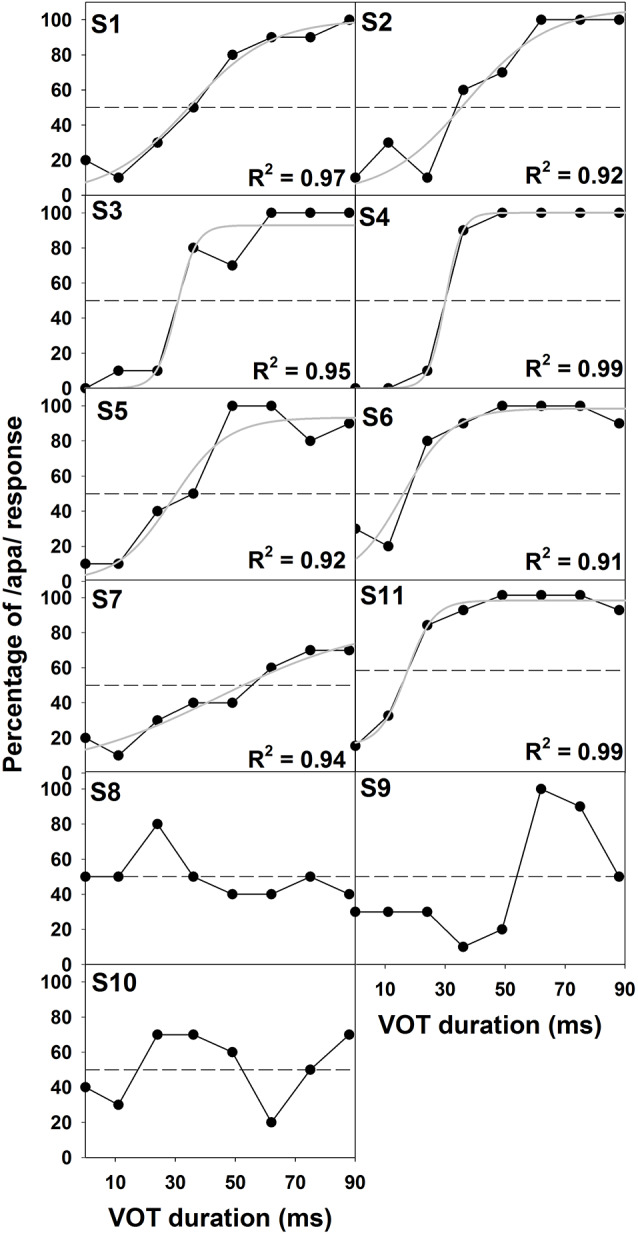
Results of behavioral categorical perception tests recorded in all subjects. In each panel, the abscissa shows VOT durations tested in this study. The ordinate indicates the percentage of /apa/ response at different VOT durations in these subjects. The subject number is indicated in the upper left corner. Also shown is the fitted psychometric function (gray lines) for results measured in S1–S7 and S11. The percentage variance that can be explained by the psychometric function (i.e., R^2^) is indicated in the lower right corner for each of these eight subjects. Results of behavioral categorical perception tests recorded in S8–S10 could not be characterized by a logistic regression function. Therefore, no psychometric function is shown for these three subjects.

**Figure 3:**
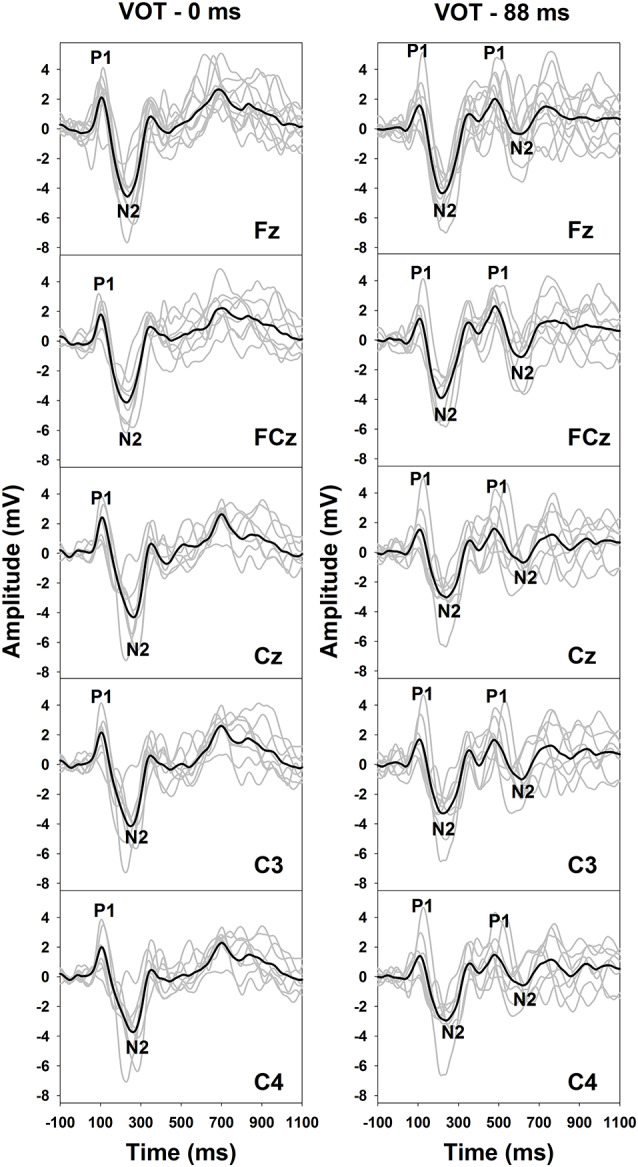
Event-related potentials (ERPs) evoked by an /aba/ token with a VOT of 0 ms (left panel) and an /apa/ token with a VOT of 88 ms (right panel) in quiet. Responses recorded at all electrode locations are shown in both panels. Gray lines indicate responses recorded in individual subjects. Black lines represent the group averaged responses. P1 and N2 peaks of the onset response are labeled for these traces. Also, P1 and N2 peaks of the auditory change complex (ACC) response are labeled for traced recorded in the 88 ms condition.

**Figure 4:**
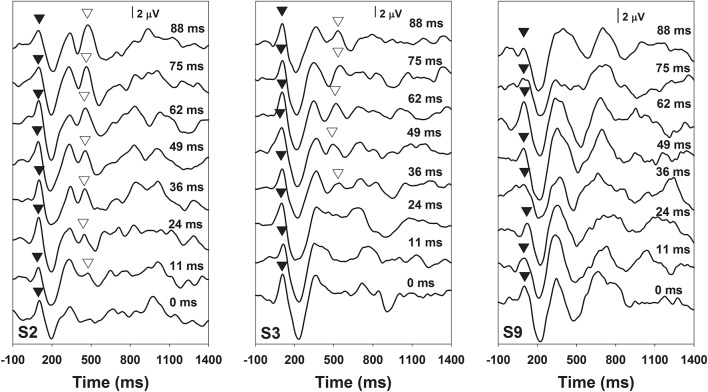
ERPs recorded at recording electrode site Fz in subjects S2, S3, and S9. The subject number is indicated in each panel. Each trace is an average of three replications recorded at each stimulation condition. Durations of VOT that were used to evoke these responses are labeled for each trace in each panel. P1 peaks of the onset and the ACC responses are indicated using filled and open triangles, respectively.

Figure 2 shows the results of behavioral categorical perception of VOT measured in all subjects. Each panel shows the percentage of trials that the speech token was perceived as /apa/ plotted as a function of VOT duration measured in one subject. In general, psychometric function fits for data recorded in S1–S7 and S11 were good, accounting for 91–99% of the variance in these data. For these eight subjects, auditory perception of these speech tokens changed from /aba/ to /apa/ as VOT duration increased. However, these results showed large individual variability. While some subjects demonstrated relatively short behavioral categorical boundaries of the /aba-apa/ continuum (e.g., 17.4 ms in S6), other subjects required relatively long VOTs for the speech token to be perceived as the /apa/ (e.g., 56 ms in S7). Also, the steepness of the fitted psychometric functions as indicated by the slope varied considerably across subjects. The slope of the psychometric function ranged from 2.7 to 16.4 percent of trial/ms with a mean of 9.2 percent of trial/ms (*SD* = 5.0). Results of a Spearman Rank Correlation test showed no significant correlation between the slope of the psychometric function and behavioral categorical boundaries in these subjects *ρ* = −0.15, *p* = 0.36). Results measured in three subjects (S8–10) showed evidence of auditory confusion in VOT perception. Specifically, auditory perception of these speech tokens did not necessarily change from /aba/ to /apa/ as the VOT increased, which indicated that the VOT cannot be accurately perceived by these three subjects. These functions do not fit a sigmoidal distribution. Therefore, the behavioral categorical boundary of the VOT could not be defined for these three subjects. For data analysis, a conservative estimate of 89 ms was used as their behavioral categorical boundaries of the VOT in this study. Figure 3 shows ERPs recorded from individual subjects (gray lines) and grand averages (black lines) recorded at five electrode sites in quiet for VOTs of 0 ms (left panel) and 88 ms (right panel). It should be noted that data shown at Fz included ERP responses recorded from all subjects and data shown at other electrode locations only include results recorded in a subgroup of eight subjects. Robust onset responses could be easily identified in these results. For responses evoked by the VCV syllable with a VOT of 88 ms, the ACC response could also be identified in addition to the onset response. Both the onset and the ACC response consist of a P1 peak followed by an N2 peak despite individual variability in amplitudes and latencies of both responses. Figure 4 shows ERP responses recorded in three subjects (S2, S3, and S9). These three subjects were selected because their results extended the entire range of objective categorical boundaries measured in this study. ERP responses were only recorded at electrode sites Fz and C3 in S9 due to insufficient subject cooperation for prolonged testing time. Therefore, only ERPs recorded at the mid-line electrode site (Fz) were shown for all three subjects. Robust onset responses were recorded in each subject at all VOT durations. Besides, ACC responses elicited by VOTs were also recorded in S2 and S3. The objective categorical boundary was 11 and 36 ms in S2 and S3, respectively. The ACC response cannot be identified in ERP responses recorded in S9. Therefore, the objective categorical boundary was determined to be 89 ms as a conservative estimate for data analysis. Results of the behavioral categorical test also showed that this subject could not perceive VOT despite the good audibility of the stimuli (Figure 2).

Effects of VOT durations on ACC amplitudes and latencies of P1 and N2 peak were evaluated using a Spearman Correlation test for results recorded in a subgroup of seven subjects (S1–S6 and S11). Responses were analyzed from the recording electrode Cz to compare our results with the published literature. Results of correlation analyses showed that P1 latencies increased as the VOT duration increased in all seven subjects (*p* < 0.05). However, the effects of the VOT duration on ACC amplitudes and N2 latencies were less consistent across subjects.

For ERPs recorded in the seven subjects who had results at all five recording locations, effects of recording locations on response amplitude, P1, and N2 latencies of the onset and the ACC responses evoked by stimuli with different VOT durations were evaluated using Friedman tests with the recording electrode location as the within-subject factor. Results showed that there were significant differences in amplitude of the onset response ($\chi_{\left(4\right)}^{2}$ = 15.93, *p* < 0.05) and the ACC ($\chi_{\left(4\right)}^{2}$ = 11.35, *p* < 0.05) recorded at different recording locations. For the onset response, results of related-sample Wilcoxon signed-rank tests showed significant differences in amplitude between results recorded at three electrode pairs [i.e., Fz vs. C4 (*p* = 0.012), Fz vs. C3 (*p* = 0.018), and Cz vs. C4 (*p* = 0.017)]. For the ACC, results of related-sample Wilcoxon signed-rank tests showed a significant difference in amplitude between results recorded at Fz and those measured at C4 (*p* = 0.036). There was no significant difference in amplitudes of the onset of the ACC recorded between any other two recording electrode locations (*p* > 0.05). Recording locations did not show significant effects on P1 latency (the onset: $\chi_{\left(4\right)}^{2}$ = 0.84, *p* = 0.93; the ACC: $\chi_{\left(4\right)}^{2}$ = 8.08, *p* = 0.09) or N2 latency (the onset: $\chi_{\left(4\right)}^{2}$ = 6.27, *p* = 0.18; the ACC: $\chi_{\left(4\right)}^{2}$ = 1.47, *p* = 0.78) of the onset of the ACC response.

**Figure 5:**
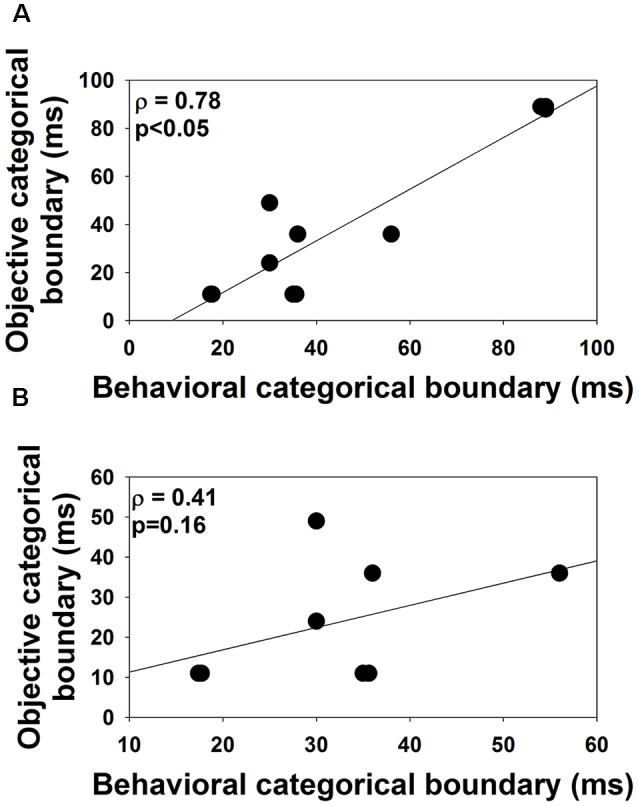
The association between the objective and the behavioral categorical boundaries of the /aba-apa/ continuum measured in this study. Panel **(A)** shows results recorded in all subjects. Panel **(B)** shows the results recorded in S1–S7 and S11. Each dot indicates results measured in one subject. Results of the one-tailed Spearman Rank correlation test are shown in the upper right corner.

VOT boundaries measured in quiet using behavioral and ERP measures are listed in Table 2. Results of the related-sample Wilcoxon Signed Rank test showed no difference between VOT boundaries measured in quiet using behavioral or ERP measures (*p* = 0.09). Excluding the results of S8–S10 did not change this statistical result. Figure 5A shows the associations between VOT boundaries measured in quiet in all subjects using these two procedures. Also shown is the result of linear regression. Results of the one-tailed Spearman Rank Correlation test showed a robust correlation between VOT boundaries estimated using these two procedures *ρ* = 0.78, *p* < 0.05). However, a careful inspection of the Figure 5A suggests that this significant correlation might have been driven by the results of S8–10. Figure 5B shows the associations between VOT boundaries measured in quiet using these two procedures in S1–S7 and S11. The association between these two measures is less strong than that shown in Figure 5A. This observation is consistent with the non-significant correlation revealed by the results of the one-tailed Spearman Rank Correlation test *ρ* = 0.41, *p* = 0.16).

**Table 2:** Objective and behavioral categorical boundaries of voice onset time (VOT) in milliseconds measured in quiet and noise.

Subject number	Behavioral categorical boundary in quiet	Objective categorical boundary in quiet	Behavioral categorical boundary in noise	Objective categorical boundary in noise
1	35	11	89	24
2	35.6	11	49.2	24
3	36	36	36	36
4	30	24	41	24
5	30	49	31	49
6	17.4	11		
7	56	36		
8	89	89	89	89
9	89	89		
10	89	89		
11	17.4	11	36.6	36

**Figure 6:**
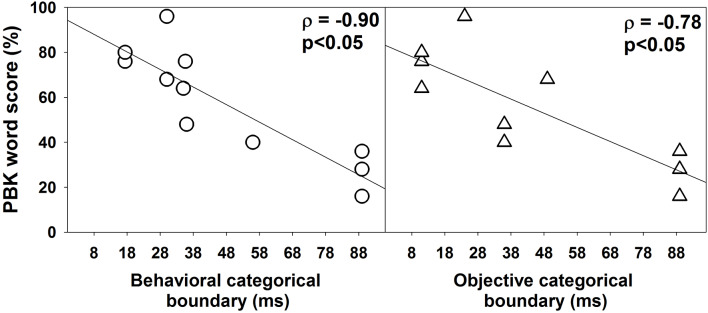
The association between phonetically balanced kindergarten (PBK) word scores and categorical boundaries estimated using behavioral and electrophysiological measures. In each panel, each symbol represents results measured in one subject. Also shown are results of linear regressions. Results of one-tailed Spearman Rank Correlation tests are shown in the upper left corner in each panel.

Figure 6 shows the association between PBK word scores, behavioral (left panel), and objective categorical boundaries of VOT (right panel) measured in quiet. Results of one-tailed Spearman Rank Correlation tests showed robust negative correlations between PBK word scores and categorical boundaries of the VOT estimated using both behavioral *ρ* = −0.90, *p* < 0.05) and objective *ρ* = −0.79, *p* < 0.05) measures.

In summary, for results measured in quiet, subjects tested in this study showed a wide range of behavioral categorical boundaries of VOT. While some subjects could not accurately perceive VOTs, other subjects required relatively short VOTs for the speech token to be perceived as the /apa/. The onset response of the speech evoked ERP was recorded in all subjects tested in this study. In contrast, not all subjects showed the ACC response. The objective categorical boundary ranged from 11 to 89 ms. Categorical boundaries of VOT measured in quiet using behavioral or ERP measures are negatively correlated with PBK word scores.

### Effect of Adding Competing Background Noise

VOT boundaries measured at an SNR of 5 dB using behavioral and ERP measures are listed in Table 2. Results of a related-sample Wilcoxon Signed Rank test showed a non-significant difference between behavioral and objective categorical boundaries of VOT measured in noise (*p* = 0.23). The correlation between categorical boundaries of VOT estimated using these two procedures was not statistically significant, as revealed by the results of a one-tailed Spearman Rank Correlation test *ρ* = −0.26, *p* = 0.28).

Inspection of Table 2 suggests that adding a competing background noise has different effects on behavioral and objective categorical boundaries of VOT. For results measured using behavioral procedures, results of a related-sample Wilcoxon Signed Rank test showed that behavioral categorical boundaries of VOT measured in noise were significantly longer than those measured in quiet (*p* < 0.05). However, there was substantial inter-subject variability. While some subjects showed significant increases in behavioral categorical boundaries (e.g., S1 and S11), other subjects demonstrated negligible changes in their behavioral categorical boundaries (e.g., S4 and S5). For results measured using objective procedures, results of a related-sample Wilcoxon Signed Rank test showed that adding a competing background noise did not significantly increase the categorical boundaries of VOT (*p* = 0.10). Similar to results measured using behavioral procedures, adding competing background noise showed mixed results across these seven subjects who were tested in both quiet and noise. While some subjects showed prolonged objective categorical boundaries (i.e., S1, S2, and S11), other subjects showed the same objective categorical boundaries regardless of the presence/absence of the background noise (i.e., S3–S5).

**Figure 7:**
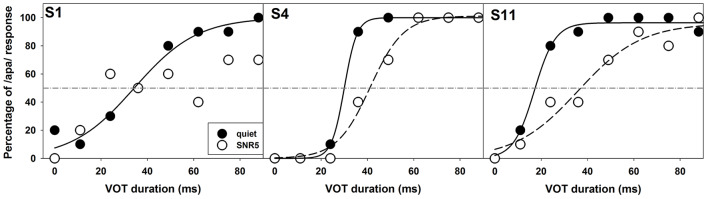
Results of behavioral categorical perception tested in quiet (filled circles) and in noise (open circles) in S1, S4, and S11. The subject number is indicated in each panel. Data collected in quiet and noise are indicated using filled and open circles. Solid and dashed lines indicate fitted psychometric functions for results measured in quiet and in noise, respectively.

**Figure 8:**
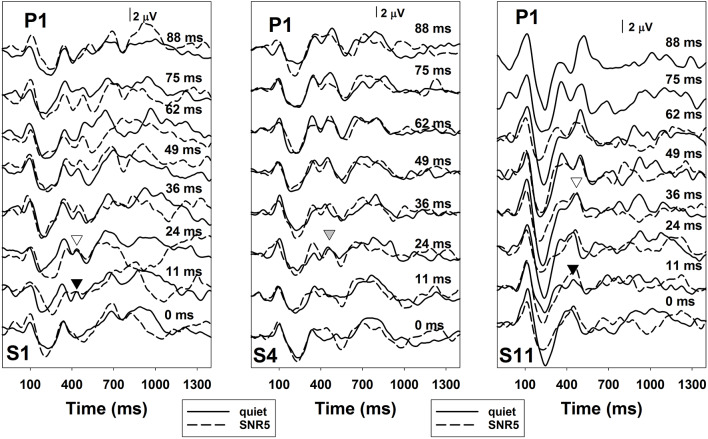
ERPs measured at recording electrode site Cz in quiet (solid lines) and in noise (dashed lines) in S1, S4, and S11. Each panel shows results recorded in each subject. The subject number is indicated in these panels. Responses measured in quiet are indicated using solid lines and results recorded at an signal-to-noise ratio (SNR) of 5 dB are indicated using dashed lines. Each trace represents an averaged response of 300 artifact-free sweeps. Black and white triangles represent the P1 of the ACC evoked at the objective categorical boundary in quiet and in noise, respectively. The gray triangle indicates the P1 of the ACC evoked by a VOT of 24 ms in S4.

The comparison of noise effects on results of ERP measures and the behavioral categorical test showed some interesting findings. While masking noise affected both neural encoding and auditory perception of VOT in some subjects (S1, S2, S4, and S11), it did not affect results recorded in other subjects (S3 and S5). Also, for these four subjects whose results were affected by masking noise, the degree of these effects was different for ERPs and behavioral auditory perception of VOT. While some subjects showed masking noise affecting both measures in a similar degree (S2 and S11), other subjects demonstrated much larger noise effects on their auditory perception than the neural encoding of VOT (S1 and S4). Figures 7, 8 show results recorded in three subjects with different effects of masking noise on these two measures. Figure 7 shows the results of behavioral categorical perception. Left, middle and right panels show results measured in S1, S4, and S11, respectively. Adding a competing noise decreased the slopes of psychometric functions in S4 and S11. Also, objective categorical boundaries increased by 11 ms in S4 and 19.2 ms in S11 with the presence of noise. Results measured in S1 clearly showed that this subject could not perceive VOT with a competing background noise despite a good categorical perception in quiet. Figure 8 shows ERPs recorded at Cz in S1, S4, and S11. Robust ERPs were recorded in all three subjects in both quiet and noise. Adding a competing background noise had different effects on the neurophysiological representation of VOT in these three subjects. For S1 and S11, filled and open triangles represent the P1 of the ACC evoked at the objective categorical boundary in quiet and in noise, respectively. Adding a competing background noise at an SNR of 5 dB in S1 and S11 increased the objective categorical boundary by 13 and 24 ms, respectively. For S4, ERP measures in quiet and noise yielded the same categorical boundary (i.e., 24 ms). The P1 of the ACC evoked by a VOT of 24 ms is indicated using a gray triangle. Comparisons between results shown in Figures 7, 8 indicate that S1 cannot perceive VOT despite a relatively good neural encoding of this timing cue at an SNR of 5 dB. For S4, adding a competing noise did not affect ERPs evoked by the VOT, but did show a small effect in auditory perception. For S11, ERPs and auditory perception of VOT were affected by a competing noise in parallel.

For each study participant, amplitudes and latencies of the onset response recorded in all VOT conditions were averaged. The calculation was conducted separately for data recorded in quiet and noise. Differences in these two variables between results measured in these two conditions were compared using related-sample Wilcoxon Signed Rank tests. Similar comparisons were conducted for the ACC results, except that the comparison only included data recorded in VOT conditions, where the ACC was recorded in both quiet and noise conditions. For the onset response, there was no significant difference in amplitude (*p* = 0.11) or N2 latency (*p* = 0.71) measured in quiet and noise. However, P1 latencies measured in noise were significantly longer than those measured in quiet (*p* < 0.05). Adding a competing noise did not show a significant effect on amplitude (*p* = 0.06), P1 latency (*p* = 0.21), or N2 latency (*p* = 0.88) of the ACC response.

In summary, adding a speech-shaped noise at an SNR of +5 dB only significantly increased the categorical boundaries of VOT estimated using behavioral procedures. The effects of competing for background noise on ERPs and auditory perception of VOT were not always in parallel and varied across subjects.

## Discussion

This study investigated the categorical perception and neural encoding of the VOT in children with ANSD in both quiet and noise conditions. Relatively long VCV stimuli that contain VOTs were used in this study as novel stimuli to better separate responses evoked by the VOT from those evoked by the syllable onset. Speech evoked ERPs were recorded in all subjects tested in this study. Onset responses recorded in all subjects were robust, which indicates the good audibility of the stimuli. In addition to the onset response, the ACC response was recorded in eight subjects at VOTs of 11 ms or longer. The P1 latency of the ACC response increased as the VOT duration increased, which is generally consistent with the existing literature (Steinschneider et al., 1999; Sharma and Dorman, 2000; Sharma et al., 2000; Tremblay et al., 2003; Frye et al., 2007; King et al., 2008; Elangovan and Stuart, 2011; Dimitrijevic et al., 2012). In three subjects (S8–S10), the ACC was not recorded at any VOTs. These three subjects also could not perceive VOT when tested using behavioral procedures. Overall, these data established the feasibility of using a relatively long VCV continuum to evaluate cortical neural encoding of VOT cues. More importantly, these results support the idea that electrophysiological measures of the ERP can be used to evaluate the neural encoding of VOT in children with ANSD.

### Results Measured in Quiet

The first hypothesis tested in this study was that the precision of the neural encoding of VOT would affect the auditory perception of VOT in children with ANSD. When all subject data is included, our results measured in quiet showed a robust correlation between categorical boundaries estimated using electrophysiological measures of ERPs and behavioral procedures in children with ANSD, which is consistent with our first hypothesis. When the results of three subjects (S8–S10) who showed the longest VOT were excluded, the trend showing the potential association between categorical boundaries of VOT estimated using these two procedures was not statistically significant. The non-significant correlation between results measured using behavioral and objective measures did not support our first hypothesis but is consistent with the literature. Specifically, in NH adults, ERPs evoked by VOTs show morphological changes in the N1 peak as VOT increases. However, the results of several studies show that these morphological changes appear to be only dependent on the acoustic properties of stimuli and independent of categorical perceptions of VOTs in NH listeners (Sharma and Dorman, 1999, 2000; Sharma et al., 2000; Elangovan and Stuart, 2011). These studies showed that the categorical pattern of the ERP response can be reliably evoked by VOTs of 40 ms or longer regardless of the subject’s categorical perception.

For subjects tested in this study, categorical boundaries measured in quiet using both electrophysiological and behavioral procedures strongly correlated with their most recently measured PBK word scores (measured 1 month prior). Subjects with longer categorical boundaries of VOT had worse speech perception performance. These data are consistent with those reported in He et al. (2015) showing the negative correlation between gap detection threshold and speech perception scores in children with ANSD. Overall, these results suggest that the ability of children with ANSD to perceive timing cues is critical for speech understanding, which is consistent with previous studies (e.g., Zeng et al., 1999; Rance et al., 2008; Starr and Rance, 2015).

### Effects of Competing for Background Noise

Several studies have shown that adding a competing background noise can have a significant negative effect on speech perception in patients with ANSD (Kraus et al., 1984, 2000; Zeng and Liu, 2006; Rance et al., 2007; Berlin et al., 2010). The underlying mechanisms for this observation are largely unknown. The second hypothesis tested in this study was that cortical encoding of VOT as assessed using ERPs in children with ANSD would be adversely affected by a competing background noise, which might account for the excessive difficulty in speech understanding with the presence of a competing noise in these patients. However, our results showed that adding a competing background noise at an SNR of 5 dB only prolonged objective categorical boundaries in three subjects (S1, S2, and S11). Neurophysiological representation of VOT was largely unaffected by the competing noise in S3, S4, and S5. More interestingly, comparisons between behavioral and objective categorical boundaries of VOT measured in noise revealed some discrepancies. For example, S4 needed longer VOT for voiceless consonant perception in noise even though neural encoding of VOT is largely unaffected by noise. Also, S1 apparently could not perceive VOT at all with the presence of background noise even though VOTs of 24 ms or longer can be accurately encoded at the central auditory system. These results suggest that underlying mechanisms for excessive speech understanding in noise may vary among patients with ANSD, perhaps in relation to varying etiologies. While disruption of neural encoding of acoustic cues can account for the difficulty in some patients, the involvement of higher-order brain functions may exist in other patients. Results of previous studies have suggested that the parallel maturation of cognitive and linguistic skills are important for speech recognition in adverse listening conditions for children (Sullivan et al., 2015; McCreery et al., 2017; MacCutcheon et al., 2019). Specifically, greater working memory capacities are associated with better speech perception in children (Stiles et al., 2012; Sullivan et al., 2015; McCreery et al., 2017; MacCutcheon et al., 2019). Possibly some children with ANSD (e.g., S1 and S4) might have declined working memory capacities, which reduced their capabilities of perceiving VOT cues. Unfortunately, due to limited testing paradigms and recording electrode sites and the lack of cognitive function evaluation, we cannot delineate underlying mechanisms for individual subjects. Further studies on these underlying mechanisms are warranted.

Our results showed that adding a competing noise prolonged P1 peak of the onset ERP response, which is generally consistent with the published literature (Whiting et al., 1998; Kaplan-Neeman et al., 2006; Billings et al., 2009, 2011, 2013; McCullagh et al., 2012; Baltzell and Billings, 2013; Kuruvilla-Mathew et al., 2015; Papesh et al., 2015). The effects of masking noise on ERP amplitudes have not been consistently reported across studies. Whiting et al. (1998) showed that masking noise did not significantly affect ERP amplitudes for responses evoked at SNRs of 0 dB or better. In our study, the presence of competing for background noise at an SNR of 5 dB did not show significant effects on ERP amplitudes, which is consistent with the results of Whiting et al. (1998). An insignificant effect of masking noise on ERP amplitudes has also been reported in other studies (e.g., Kuruvilla-Mathew et al., 2015). However, changes in ERP amplitudes with the presence of masking noises have been observed and reported (e.g., Alain et al., 2009; Parbery-Clark et al., 2011; Billings et al., 2013; Papesh et al., 2015). The discrepancy among results reported in these studies is not entirely clear. Papesh et al. (2015) recently showed that ERP amplitudes are affected by stimulus presentation factors and response component of interest. For example, adding a low-level background noise could enhance the N1 peak at fast presentation rates, but decrease the P1 and the P2 peak regardless of the presentation rate. The amount of enhancement in N1 amplitude depends on the noise level, with higher noise levels resulting in smaller enhancement. Slower presentation rates (e.g., 2 Hz) lead to reductions in N1 amplitudes in noise. Using binaural presentation increases the amplitude of the P1 and the P2 peak, but does significantly affect the N1 peak. Therefore, differences in stimulus presentation factors and methods used to quantify ERP amplitudes among these studies might, at least partially, account for the discrepancy in reported study results. The effects of masking noise on ACC responses have not been systematically evaluated in human listeners. Further studies are needed to confirm our findings.

### Study Limitations

This study has several potential limitations. First, due to time constraints, there were only a limited number of VOTs tested in this study. In three study participants (S8, S9, and S10), the ACC was not recorded at the longest VOT tested (i.e., 88 ms). For these participants, the objective category boundary was conservatively estimated to be 89 ms and used in data analysis. This conservative approach should not have changed the overall direction of the correlation between the objective and behavioral categorical boundaries of the VOT or between categorical boundaries of the VOT and PBK word scores. However, it could have reduced the magnitude of these correlations because the actual objective categorical boundary was longer than 89 ms in these participants. Second, due to the challenges of recruiting children with ANSD and normal cognitive function who could complete multiple testing sessions scheduled on the same day, only 11 subjects were tested in this study, which might have limited the statistical power of this study. Third, even though maximum efforts were implemented to hold other acoustic/phonetic properties constant when VOT was manipulated in this study, the stimulus might still include spectral cues (Figure 1). Therefore, the results of this study might not only reflect temporal processing capacities in children with ANSD. Finally, whereas ERPs evoked by the VOT evaluate passive neural encoding of VOT cues, the behavioral categorical perception test requires active subject participation and relies on the subject’s cognitive function. Therefore, the possibility that these two measures may assess different mechanisms underlying speech perception cannot be excluded. Due to these study limitations, the results of this study need to be interpreted with caution.

### Contributions to the Literature

Despite these study limitations, the results of this study made three contributions to the literature. First, the results of this study established the feasibility of using a relatively long VCV continuum to evaluate cortical neural encoding of VOT cues. This is a novel stimulus for ERP measures. Second, these results supported the idea that electrophysiological measures of the ERP can be used to evaluate the neural encoding of VOT in children with ANSD, which addressed a knowledge gap in the field. Finally, data from this study indicated that the underlying mechanisms for excessive speech understanding in noise for patients with ANSD are heterogeneous. Unfolding these mechanisms will require assessing a large group of patients with ANSD using a comprehensive testing battery, including neurocognitive assessments, electrophysiological, and behavioral testing procedures.

## Conclusion

VCV continua can be used to evaluate behavioral identification and the neural encoding of VOT in children with ANSD. Categorical boundaries of VOT estimated using behavioral measures and ERP recordings are closely associated in quiet. Therefore, electrophysiological measures of the ERP can be used to evaluate the neural encoding of VOT in children with ANSD. The results of these measures also show a relationship with speech perception scores in subjects tested in this study. Underlying mechanisms for excessive speech perception deficits in noise may vary for individual patients with ANSD.

## Data Availability Statement

The datasets generated for this study are available on request to the corresponding author.

## Ethics Statement

The studies involving human participants were reviewed and approved by The Institutional Review Board (IRB) at The University of North Carolina at Chapel Hill. Written informed consent to participate in this study was provided by the participants’ legal guardian/next of kin.

## Author Contributions

TM and PB participated in data collection, data analysis, results interpretation, and are accountable for all aspects of this study. They also provided critical revision and approved the final version of this article. JS designed the speech stimuli used in this study, provided critical comments, and approved the final version of this article. SH designed and is accountable for all aspects of this study, as well as drafted and approved the final version of this article.

## Conflict of Interest

The authors declare that the research was conducted in the absence of any commercial or financial relationships that could be construed as a potential conflict of interest.
